# Emergency Medical Teams Interventions due to Cardiovascular Diseases in 2018: Polish Regional Observational Study

**DOI:** 10.3390/medicina57020139

**Published:** 2021-02-04

**Authors:** Klaudiusz Nadolny, Magdalena Wierzbik-Strońska, Jerzy R. Ładny, Beniamin O. Grabarek, Oliwia Warmusz, Dariusz Boroń, Aleksander Ostenda

**Affiliations:** 1 Faculty of Medicine, University of Technology in Katowice, 40-555 Katowice, Poland; klaudiusznadolny3@gmail.com (K.N.); warmuszoliwia@gmail.com (O.W.); aleksander.ostenda@wst.com.pl (A.O.); 2 Department of Emergency Medical Service, Strategic Planning University of Dabrowa Gornicza, 40-555 Dąbrowa Górnicza, Poland; 3 Department of Emergency Medcine, Medical University of Bialystok, 15-295 Białystok, Poland; jerzyladny3@gmail.com; 4 Department of Histology, Cytophysiology and Embryology, Faculty of Medicine, University of Technology in Katowice, 41-800 Zabrze, Poland; bgrabarek7@gmail.com (B.O.G.); dariusz@boron.pl (D.B.); 5 Department of Gynecology and Obstetrics with Gynecologic Oncology, Ludwik Rydygier Memorial Specialized Hospital, 31-826 Kraków, Poland

**Keywords:** Silesian Voivodeship, gold hour, cardiovascular diseases, Medical Emergency Team

## Abstract

*Background and objectives:* The goal of this work was to assess the interventions for cardiovascular causes (ICD-10: I) and analyze the time between the request for intervention and the arrival of the Medical Emergency Team realized by the Voivodeship Rescue Service in Katowice in the period between 1 January 2018 to 31 December 2018. *Materials and Methods:* Analysis of the characteristics of the interventions was completed based on the information contained on the dispatch order cards and medical emergency services. Statistical analysis was done using the Chi-square test (*p* < 0.05). *Results:* Out of 211,548 cases, 26,672 were associated with cardiovascular diseases. It can be observed that the large majority of interventions took place in urban areas (89.98%; 23,998 cases), whereas only 11.02% took place in rural areas (2674 cases). The most common cause for medical interventions being made by the Medical Emergency Team was primary hypertension—11,649 cases. The average arrival time to urban areas was 9 min and 12 s ± 3 min and 54 s, whereas for rural areas it was 11 min and 57 s ± 4 min and 32 s (*p* < 0.05). *Conclusions:* It can be observed that the Medical Emergency System in Katowice operates accordingly with the intentions of the legislator. The obtained data also indicates that there is a high societal awareness of the residents about the purpose of the Medical Emergency Team.

## 1. Introduction

The process of creating the Emergency Medical Services began in the 1990s from the creation of the Integrated Medical Rescue. However, starting only from 25 July 2001 the first act about the Emergency Medical Services was created [1]. Throughout the following years, the assumptions of the act were revised, which led to the creation of the act currently in place from the 8 September 2006 [2,3]. The creation of a formalized structure in the form of a system based on the interdependencies of the individual components that make it up, such as people, products, and services, which are all connected with the implementation of one common goal was a key undertaking that conditioned the saving of human life. The main goal of the Medical Rescue System is guaranteeing help in sudden situations that directly threaten the life of a person [4,5]. Included in the Emergency Medical Services are Medical Emergency Teams (ambulances; air ambulances; water ambulances) and also Hospital Emergency Wards [6]. The primary task of the Medical Emergency Team is granting help to the victim on site of the incident, and if it is advisable, to also transport the victim to the appropriate reference unit in the shortest time possible [7]. The second, incredibly important units are the Hospital Emergency Wards, which are responsible for carrying out the initial diagnosis as well as treating the person in the necessary range, which is especially important in sudden life-threatening situations [8]. In reference to the Emergency Medical Service system, an incredibly important term is the effectiveness of action, defined as the correct action being done in the correct method, where effectiveness and efficiency are key. It is also worth noting the two critical elements in the functioning of the Emergency Medical Services in Poland [9,10]. One of which is highlighting the role of the medical distributor, who, based on the information they gather from the interview they carried out through telephone communication and also on their own knowledge and subjective instinct decides, whether an intervention by the Medical Emergency Team is or is not necessary [11,12,13]. A second factor that determines the effectiveness of the system is the time taken between the moment an incident was reported (accident) to the moment the Medical Emergency Team arrives at the incident site. Therefore, a conversion factor is adopted in this regard, that on average every 2 min, a distance of at least 1 km has to be covered [14].

One of the causes of undertaking an intervention by the Medical Emergency Team were reports due to cardiovascular diseases, which are a wide range of diseases according to the International Classification of Diseases—ICD-10 [15]. It is estimated that in Poland, approximately 100 people die each day due to heart failure, which constitutes around 20% of all deaths due to cardiovascular problems. Moreover, an unsettling fact is that one in three male deaths and one in 10 female deaths are due to cardiovascular diseases for people above 64 years of age, which is the group of people most active professionally [16,17].

The goal of this work was to assess the interventions for cardiovascular causes (ICD-10: I) and analyze the time between the request for intervention and the arrival of the Medical Emergency Team realized by the Voivodeship Rescue Service in Katowice in the period between 1 January 2018 to 31 December 2018.

## 2. Materials and Methods

Firstly, from all the accepted calls by the Voivodeship Ambulance Service, the calls in which the medical dispatcher found it necessary for intervention on-site were selected. For this type of study (survey), approval of the bioethics committee is not required. In the second stage, the analyzed interventions were narrowed down based on identification criteria, according to the International Classification of Diseases, ICD-10. The identifications made using the code I were selected, which covers cardiovascular diseases. Next, the information contained in the “Emergency ambulance dispatch order card” was imported into an Excel calculatory spreadsheet, and afterward, statistical analysis was conducted based on the licensed version of the STATISTICA 13 PL program (StatSoft, Cracow, Poland). The analyzed data was then split based on identification, the intervention site (urban; rural) as well as sex (male; female), and also the way the intervention was completed. In this work, we also present the time that passed from the moment the call was received to the time the Medical Emergency Team arrived at the site. In the statistical analysis, the Chi-square test was used, with the statistical significance threshold adopted at *p* < 0.05.

## 3. Results

Based on the shared medical documentation, it was determined that interventions made by the Medical Emergency Team due to cardiovascular disease were 12.6% (26,672 cases) of all the completed interventions. The total number of all interventions in 2018 totaled 211,548. It can be observed that the large majority of interventions took place in urban areas (89.98%; 23,998 cases), whereas only 11.02% took place in rural areas (2674 cases). The three most common causes for interventions being made by the Medical Emergency Team included: primary hypertension—11,649 cases; stroke, not specified as hemorrhage or infarction—3740 cases; atrial fibrillation and flutter—2473 cases. In Table 1, the 10 most common causes for the emergency interventions are presented, while less common causes are grouped under “other causes”.

Afterward, how these interventions were concluded by the Medical Emergency Team was assessed. The most common decision was for the patient to be directly transported and received by the hospital emergency department or emergency room (totaling 16,465 cases, which is equal to 61.7% of all total cases). In turn, in the case of 8732 calls (29.18%), help was granted on-site of the intervention, without the need to continue diagnostics and treatment in hospital. The statistical assessment indicated the occurrence of statistical significance (Table 2).

In the last part, the time that passed between the call was received and the arrival of the Medical Emergency Team to the intervention site. The average arrival time to urban areas was 9 min and 12 s ± 3 min and 54 s, whereas in rural areas it was 11 min and 57 s ± 4 min and 32 s (*p* < 0.05).

## 4. Discussion

Cardiovascular diseases constitute the first most common cause of death worldwide; the same tendency was also noted in Poland [18]. Due to this, they form a huge challenge for the Medical Services, Emergency Medical Services as well as for the state, whose primary responsibility is guaranteeing the correct functioning of the system in sudden life-threatening situations [19]. According to the knowledge of the authors, the comparison of the trip characteristics made by the Medical Emergency Team within the territory of the Silesian Voivodeship presented as part of this work is the first of this sort of analysis. This type of analysis seems fully reasonable, as they allow for the assessment of how the organized Emergency Medical Service is used by its users (reporters), and additionally the societal awareness about the purpose of the system itself. Moreover, it also indicates the further decisions made in the given situations, which allows for determining the strengths and weaknesses of the system, and therefore, gives the ability to improve the system further [12,20,21,22]. Furthermore, such analyses are a valuable resource for developing preventive programs, indicating the target recipient group, and thanks to this, there is the possibility to create a campaign that will be met with a positive societal response [23,24].

Based on the obtained data, it was determined that decidedly, more often cardiovascular diseases were identified in men more than in women. This indicates that risk factors predisposed to the appearance of cardiovascular disease do not differ in a significant way between men and women. Simultaneously, however, it is worth noting that individual factors may have different severity in affecting people of both sexes [25,26,27,28]. For example, diabetes contributes 6–7 times more often to the development of ischemic disease in women, whereas only 2–3 times in men [29,30]. The most common reported cardiologic problem was primary hypertension. In 65% of accepted cases, the decision was made that it was necessary to grant further specialist healthcare in the Hospital Emergency Ward. Whereas, in nearly ⅓ of cases the help was granted on-site. This suggests that the majority of primary hypertension cases could constitute a direct threat to the life and health of a person, and furthermore, shows that the decision and assessment made by the distributor were correct [13]. Indirectly, this may also indicate the ability that the distributor possesses throughout the initial interview, in collecting key information about the health state of the patient, as well as the ability of the person reporting the situation to describe it to ask for help [2,13]. A similar tendency was observed for the second most common cause for calls for the Medical Emergency Team, which is atrial fibrillation and flutter as well as heart failure, which constitutes the third most common cause for interventions being made by the Medical Emergency Team.

However, a key element of the Emergency Medical Service system is also the time taken between accepting a call by the distributor and the arrival of the Medical Emergency Team. According to the act currently in effect about the Emergency Medical Services in regions in which over 10,000 residents are located, the time of arrival for the Medical Emergency Team in urban areas should not be more than 8 min, whereas in rural areas it should not be longer than 15 min [2]. A significant fact also seems to be that in the period the act was being created, it was decided to move towards shortening the maximum allowed time for arrival. First, in the act from 2001, it was decided that a Medical Emergency Team should arrive in urban areas in 20 min and to rural areas in 30 min [1,2]. The changes that were made by the legislator between 2001 and 2006 aimed to use the so-called “golden hour” in the best way possible, as it could decide whether the victim survives or not [31]. The data obtained by us alongside the existing recommendations indicates a shorter than required time for arrival to the victim in urban areas at 9 min and 12 s ± 3 min and 54 s, whereas for rural areas the time taken is 11 min and 57 s ± 4 min and 32 s [2]. It can also be determined that the average arrival time by the Voivodeship Ambulance Service in Katowice is close to the time noted by other teams, such as the Voivodeship Ambulance Service in Lublin which averaged out to be 8.55 ± 5.16 min [20], whereas in the Otwock county the average time was 9.39 ± 6.87 min [32].

## 5. Conclusions

In conclusion, it can be observed that the Emergency Medical Services in Katowice function according to the intentions of the legislator. The obtained data also indicates a high societal awareness about the correct functioning and purpose of the Medical Emergency Team.

## Figures and Tables

**Table 1:** The characteristics of the injuries to which a trip by a Medical Rescue Team was completed in 2018.

ICD-10 CODE	Name of Disease	Sex	Number of Cases in a Village	Number of Cases in a City	*p* < 0.05
I10	Primary hypertension *n* = 11,649	Female	700	7680	*p* = 0.0001
		Male	350	2905	
I64	Stroke, not specified as hemorrhage or infarction *n* = 3740	Female	201	1780	*p* = 0.8850
		Male	181	1578	
I48	Atrial fibrillation and flutter *n* = 2473	Female	146	1413	*p* = 0.1424
		Male	111	884	
I46	Cardiac arrest *n* = 1533	Female	57	480	*p* = 0.7906
		Male	100	896	
I50	Heart failure *n* = 1448	Female	93	567	*p* = 0.032
		Male	82	706	
I21	Acute myocardial infarction *n* = 886	Female	36	250	*p* = 0.4634
		Male	64	536	
I95	Hypotension *n* = 771	Female	3	427	*p* = 0.096
		Male	7	334	
I49	Other cardiac arrhythmias *n* = 653	Female	29	339	*p* = 0.1035
		Male	29	339	
I47	Paroxysmal tachycardia *n* = 625	Female	35	303	*p* = 0.4673
		Male	35	252	
I20	Unstable angina *n* = 431	Female	36	250	*p* = 0.2936
		Male	64	536	
-	Other causes *n* = 2364	Female	163	1092	*p* = 0.0899
		Male	170	939	

**Table 2:** Reasons for medical interventions of the Voivodeship Emergency Medical Teams in Katowice in 2018.

Form of Conclusion	Rural	Urban
Other than aforementioned	74 (2.8%)	581 (2.4%)
Medical emergency operations abandoned	46 (1.7%)	528 (2.2%)
The person who was helped was directly transported and received by the hospital organizational unit	11 (0.4%)	177 (0.7%)
The person who was helped was directly transported and received by the hospital emergency department or emergency room	1593 (59.6%)	14,872 (62.1%)
The person who was helped was not transported to the hospital emergency department or emergency room	949 (35.5%)	7783 (32.5%)

## Data Availability

Ethical review and approval were waived for this study, due to the retrospective nature of the study and does not bear the characteristics of a medical experiment (Decision of the Bioethical Committee of the University of Technology in Katowice, no. 5/2020).
